# Serum Ferritin Levels in Severe Obstructive Sleep Apnea

**DOI:** 10.3390/diagnostics13061154

**Published:** 2023-03-17

**Authors:** Christopher Seifen, Johannes Pordzik, Tilman Huppertz, Berit Hackenberg, Cornelia Schupp, Christoph Matthias, Perikles Simon, Haralampos Gouveris

**Affiliations:** 1 Sleep Medicine Center, Department of Otolaryngology, Head and Neck Surgery, University Medical Center Mainz, 55131 Mainz, Germany; 2 Department of Sports Medicine, Disease Prevention and Rehabilitation, Johannes Gutenberg University, 55099 Mainz, Germany

**Keywords:** obstructive sleep apnea, OSA, serum ferritin, ferritin, iron metabolism, inflammation, CRP, obesity, BMI

## Abstract

Obstructive sleep apnea (OSA) has been associated with various acute and chronic inflammatory diseases, as has serum ferritin, an intracellular iron storage protein. Little is known about the relationship between severity of OSA and serum ferritin levels in otherwise healthy subjects. In this study, all polysomnographic recordings, serum levels of ferritin, C-reactive protein (CRP), and hemoglobin, as well as patient files from 90 consecutive, otherwise healthy individuals with suspected OSA who presented to a tertiary sleep medical center were retrospectively analyzed. For comparison, three groups were formed based on apnea–hypopnea index (AHI; none or mild OSA: <15/h vs. moderate OSA: 15–30/h vs. severe OSA: >30/h). Serum ferritin levels were significantly positively correlated with AHI (r = 0.3240, *p* = 0.0020). A clear trend of higher serum ferritin levels was found when patients with severe OSA were compared to those without or with mild OSA. Serum CRP and serum hemoglobin levels did not differ significantly among OSA severity groups. Age and body–mass index (BMI) tended to be higher with increasing OSA severity. The BMI was significant higher in patients with severe OSA compared to those without or with mild (*p* < 0.001). Therefore, serum ferritin levels may provide a biochemical surrogate marker for OSA severity.

## 1. Introduction

Obstructive sleep apnea (OSA) is the most common type of sleep-disordered breathing with increasing prevalence [1]. Upper airway collapsibility during sleep is a major feature of OSA pathogenesis. Even when respiratory effort is still present, apneas or hypopneas caused by airway obstruction occur repeatedly [2]. Being overweight is the strongest risk factor for OSA [3]. Furthermore, OSA prevalence increases with growing age and is more frequently seen in males [4,5]. Previous studies have shown that OSA is related to risk of glaucoma [6], non-alcoholic hepatic steatosis [7], unfavorable oncologic outcomes following therapy for head and neck squamous cell carcinoma [8], coronary artery disease [9], stroke [10], arterial hypertension [11], and other adverse effects [12]; therefore, OSA is recognized as an important public health issue.

In general patient care, serum ferritin is part of routinely ordered blood tests, and plays an important role in the evaluation of anemia. Ferritin is an intracellular storage protein that binds iron, and its intracellular concentration regulates the production of serum ferritin [13]. Serum ferritin levels have been linked to the presence or severity of different chronic inflammatory diseases in which iron dysregulation clearly plays a major role, e.g., arterial hypertension [14], type 2 diabetes [15], coronary artery disease [16], metabolic syndrome [17], and many others [18]. Moreover, serum ferritin is nonspecifically elevated in acute infectious and tumor diseases; thus, serum ferritin is widely recognized as an acute phase reactant and a surrogate marker of inflammation [13]. Apart from adult medicine, elevation of serum ferritin in uncontrolled inflammation has also been observed in pediatric practice, so its elevation is used as a biomarker in various clinical scenarios [19]. Pathologically elevated serum ferritin levels, as seen in hemochromatosis, can cause a variety of symptom constellations. In addition to joint complaints, common symptoms include abdominal pain, hair loss, fatigue, decreased libido or weight loss [20,21].

The majority of OSA patients suffer from very common comorbidities such as arterial hypertension, metabolic syndrome, type 2 diabetes, or coronary artery disease. Hence, a positive correlation between OSA severity and serum ferritin levels appears plausible; however, few studies have addressed this question, with conflicting results. Therefore, the purpose of the present research was to investigate any possible linkage of serum ferritin levels with the severity of OSA. We hypothesized that intermittent hypoxia, the hallmark pathophysiological effect of OSA at the peripheral tissue level, contributes to the increase in serum ferritin levels with increasing severity of OSA. To this end, only otherwise healthy subjects with a clinical suspicion of OSA have been included in the present study to minimize important confounding factors possibly altering the complex iron metabolism, such as very common concomitant inflammatory diseases and other pathological conditions such as anemia, neoplastic disease, or recent inflammatory conditions.

## 2. Methods

### 2.1. Study Participants and Data Collection

We searched the databased of our sleep laboratory (which is part of a tertiary university medical center) from January 2020 to January 2022 for all patients whose clinical complaints were suggestive of underlying OSA and who underwent polysomnography (PSG) for the first time. All patients who participated in the study were screened for OSA due to history of snoring, apneas, or daytime sleepiness, or a combination of those; in other words, OSA screening was based on patient-reported sleep-related symptoms rather than routine health assessment or high-risk screening. A licensed technician ensured that each PSG was performed correctly overnight, and evaluation was performed by an ENT physician according to standard guidelines (American Academy of Sleep Medicine (AASM) [22]).

For this study, the medical files of all patients were screened for baseline characteristics, e.g., age, body–mass index (BMI), and sex. Similarly, we looked for chronic diseases (e.g., type 2 diabetes, arterial hypertension, pulmonary disease, cardiovascular disease, or chronic mental health disorders), recent infections, malignancies, and daily or regular use of medications of all types.

Only adult patients without any chronic diseases, current infections, history of malignancies, or daily or regular use of any type of medication were included in the present study. In other words, only patients who could be defined as healthy based on their records before the PSG exam were included in the study. In addition, we excluded individuals with central apnea, periodic breathing, or other types of sleep-related breathing disorders.

We analyzed the PSG recording of each patient for selected parameters (in alphabetical order):

− AHI-apnea–hypopnea index: apneas and hypopneas/h;
− AI-apnea index: apneic events/h;
− ARI-arousal index: arousal/h);
− HI-hypopnea index: hypopnea events/h;
− ODI-oxygen desaturation index: oxygen desaturation events (≥4%)/h;
− Percentage of N3 sleep (slow-wave sleep);
− Percentage of REM sleep;
− PLM-total number of periodic limb movements;
− SI-snoring index: snoring events/h;
− TST-total sleep time in minutes; and
− t90-percentage of oxygen desaturation lower than 90%.

Blood samples collected in the morning hours after the PSG exam (usually after 12 h of fasting) were analyzed for serum ferritin levels (ng/mL), serum C-reactive protein (CRP) levels (mg/L), and serum hemoglobin levels (g/dL).

For further investigation three groups were formed based on OSA severity:

(1) all male and female patients with AHI <15/h (“none/mild”);
(2) all male and female patients with AHI 15–30/h (“moderate”);
(3) all male and female patients with AHI >30/h (“severe”).

### 2.2. Statistical Analysis

Statistical analysis was performed using SPSS Statistics, version 23 (IBM Corp., Armonk, NY, USA), and JMP (SAS Institute, Cary, NC, USA). Descriptive statistics were used for presenting OSA characteristics, anthropometry, and laboratory values. Variables were described using mean and standard deviation (SD), or median and interquartile range (IQR) where applicable. Since only “age” showed normal distribution, log-normalization was applied for those values (BMI, ferritin, CRP, and AHI) where Pearson correlation analysis, stepwise logistic regression analysis and subsequent multifactorial analysis of variance was applied. We considered the results significant when the *p*-value was <0.05 (*), *p* < 0.01 (**), and *p* < 0.001 (***). Graphical illustration was performed using GraphPad Prism version 5.01 (GraphPad Software, Boston, MA, USA). Boxplots were used in the figures presenting median and IQR.

For investigation of factors that contribute to AHI, we employed a two-step procedure. We first computed a stepwise feed-forward logistic regression analysis. To ensure stringent inclusion criteria, we fed the model only with those data that showed a significant correlation with AHI. Only age, and the log-normalized values for BMI, ferritin, and CRP reached a significance level < 0.05. For entering a single of these variables into the regression equation we set again the level of entry to 0.05 and only ferritin and age were able to enter the model. These factors were then used to compute a logistic regression analysis.

### 2.3. Ethical Statement

Consent to use the data was given by all patients. All data were analyzed anonymously. The study methods complied with local research practices and data protection. The study was performed in accordance with the Declaration of Helsinki.

## 3. Results

### 3.1. The Study Population

A total of 432 patients underwent polysomnography for the first time during the period studied. A total of 342 patients had to be excluded because they were under 18 years of age, had chronic diseases, current infection, history of malignancies, daily/regular use of any type of medication, no OSA typical sleep-related breathing pattern, or double measurement at different time points. A total of 90 patients and their data sets were included for assessment in the present study. Group “none/mild” contained 30 patients (8 (26.7%) female), the age was 19–66 years (42.11 ± 12.99 years) and BMI was 18–44 kg/m^2^ (25 (23–27.3) kg/m^2^). A total of 31 patients (7 (22.6%) female) met inclusion criteria for group “moderate”. Age was 28–65 years (44.21 ± 11.48 years) and BMI was 22–42 kg/m^2^ (28 (26–30) kg/m^2^). Group “severe” contained 29 patients (2 (6.9%) female), the age was 35–60 years (48.06 ± 6.92 years) and BMI was 22–40 kg/m^2^ (30 (26.5–32.5) kg/m^2^). All baseline characteristics of the study group are shown in Table 1.

### 3.2. Between Group Comparison of Polysomnographic Parameters

In this initial analysis, we compared sleep parameters of the three study groups “none/mild”, “moderate”, and “severe”. All investigated respiratory parameters, as AHI, AI, and HI were significantly higher in group “moderate” compared to group “none/mild” (*p* < 0.001, *p* < 0.01, and *p* < 0.001, respectively), in group “severe” compared to group “moderate” (*p* < 0.001, *p* < 0.001, and *p* < 0.05, respectively), and in group “severe” compared to group “none/mild” (*p* < 0.001 for all parameters). Evaluation of pulse oximetry measurements showed that ODI and t90 were significantly elevated in group “moderate” compared to group “none/mild” (*p* < 0.001, and *p* <0.05, respectively), in group “severe” compared to group “moderate” (*p* < 0.001, and *p* < 0.01, respectively), and in group “severe” compared to group “none/mild” (*p* < 0.001 for both parameters). PLM showed a tendency of higher values according to OSA severity, with significantly higher values in group “severe” compared to group “none/mild” (*p* < 0.05). Comparison between groups revealed no significant differences in TST. N3 sleep (%) was significantly lower in group “severe” compared to group “none/mild” (*p* < 0.01) and compared to group “moderate” (*p* < 0.01), while comparison between groups revealed no significant differences in REM sleep (%). The ARI was significantly elevated in group “severe” compared to group “none/mild” (*p* < 0.001) and compared to group “moderate” (*p* < 0.01). All above mentioned sleep parameters are shown in Table 2.

### 3.3. Ferritin, C-Reactive Protein, and Hemoglobin Serum Levels

**Figure 1:**
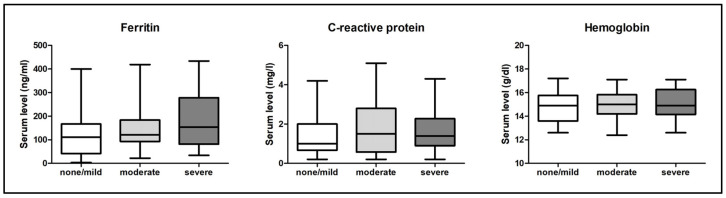
Ferritin, C-reactive protein, and hemoglobin serum levels among the three different patient groups. “none/mild”—all male and female patients with no or mild form of obstructive sleep apnea (OSA); “moderate”—all male and female patients with moderate form of OSA; “severe”—all male and female patients with severe form of OSA. Within the boxplots, median and IQR are presented.

**Figure 2:**
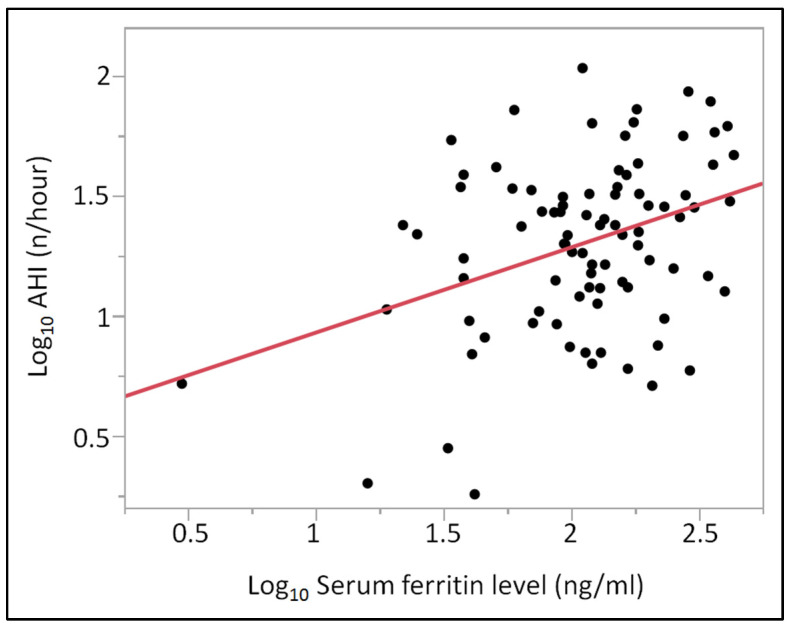
Correlation analysis between log_10_-transformed apnea–hypopnea index and log_10_-transformed serum ferritin level in the entire study cohort. Abbreviation: AHI-apnea–hypopnea index. Pearson correlation identified a significant positive correlation between AHI and serum ferritin level (r = 0.3240, *p* = 0.0020).

Analysis of the serum ferritin revealed a clear trend of elevated levels in group “severe” compared to group “none/mild”. In addition, a higher tendency of serum ferritin level was found in group “moderate” compared to group “none/mild”, but without statistical significance. Pearson correlation analysis identified a significant positive correlation between AHI and serum ferritin level (r = 0.3240, *p* = 0.0020), even after adjusting for the potentially confounder “age” (r = 0.3639, *p* = 0.0032). Median serum ferritin levels were 136 (92–194) ng/mL in men and 60 (35.5–131) ng/mL in women (*p* < 0.01). Contrarily, analysis of CRP or hemoglobin serum levels revealed no significant differences between patients with none/mild, moderate, or severe OSA. No patient suffered from acute or chronic inflammation and no patient had anemia. All above mentioned ferritin, CRP, and hemoglobin serum levels are shown in Table 3 and presented in Figure 1. The correlation analysis between AHI and serum ferritin level is presented in Figure 2.

Although sex did not have a statistically significant association with AHI, we found significant correlations for age, BMI, ferritin and CRP as frequently described in the literature (Table 4). To assess which of these factors contributes to the variance in AHI, we conducted a stepwise feed-forward logistic regression analysis. These factors were then used to compute a logistic regression analysis. In the logistic regression analysis, only ferritin achieved a statistical power level of 0.80, and its influence on AHI was computed adjusted for age (statistical power level < 0.50).

## 4. Discussion

In the present study, we demonstrated that serum ferritin levels tended to be higher with increasing severity of OSA in otherwise healthy subjects. A quite relevant difference was found when patients with severe OSA were compared to those without or with mild OSA. Accordingly, we demonstrated a significant positive correlation between AHI and serum ferritin levels. In contrast, serum CRP levels and serum hemoglobin levels did not differ significantly between groups with different OSA severity based on AHI. In our study cohort, age and BMI tended to be higher when OSA severity (as depicted by the AHI) increased, with quite significant differences for patients without or with mild OSA versus severe OSA.

Our results are in accordance with a study by O’Brien et al. in 80 patients with OSA, where a significant association between higher serum ferritin levels and OSA was described [23]. The authors did adjustments for age, sex, and BMI, but not for possibly confounding chronic diseases, recent infection, malignancies, or medication, which could have affected their reported results. As a possible explanation the authors discussed the elevation of serum ferritin in the context of increased inflammation in patients with severe OSA. Other authors have previously advocated an enhanced production of red blood cells and increased hematocrit levels in patients with OSA [24]. However, in our study, after carefully controlling for the various relevant confounders, we provide evidence that serum CRP and hemoglobin levels, which are markers of systemic inflammation and anemia, respectively, were not affected by the severity of OSA. Regarding serum ferritin as an inflammatory marker, it could be argued that its elevation in patients with severe OSA in our cohort might be due to (or confounded by) the presumed stronger effect of obesity in this group than to OSA itself. However, this argument may be rather attenuated since comparable CRP levels in the different severity groups in our cohort were found. Another study by Ming et al. retrospectively compared 270 bariatric candidates and demonstrated that serum ferritin levels were significantly elevated in the moderate/severe OSA group compared to the no/mild OSA group [25].

Our results are contradictory to what was described by Thorarinsdottir et al. among 796 subjects with OSA that were part of the Icelandic Sleep Apnea Cohort [26]. In their study, OSA patients were compared to 637 randomly chosen Icelanders who participated in an epidemiological study. The authors proved significantly higher serum ferritin levels in OSA males and a trend in the same direction for OSA females. However, after adjusting for age, BMI, smoking history, hypertension, cardiovascular disease, and type 2 diabetes this difference in serum ferritin levels by OSA was no longer found. In addition, the authors found no significant associations between serum ferritin levels and OSA severity, both unadjusted and after adjusting for relevant confounders. Of note, Thorarinsdottir et al. found that serum ferritin levels were significantly higher in OSA males than OSA females [26], an observation that is also true for our cohort. Another study by Abakay et al. compared 44 patients with OSA to 46 controls and found no significant correlation between serum ferritin levels and OSA [27].

The number of studies on the influence of OSA on serum ferritin levels is quite limited. To our knowledge, our study is the first to provide evidence on serum ferritin levels in a large number of carefully selected, otherwise healthy participants who were first tested for OSA based on clinical sleep-related symptoms. Our study has the advantage that patients with any diseases that may have altered serum ferritin levels such as chronic diseases, malignancies, iron deficiency, hemochromatosis, or dysthyroidism were excluded from the study population. Moreover, measurement and evaluation of serum CRP and serum hemoglobin levels could also help to uncover undiagnosed or unreported inflammatory diseases or subclinical anemia to exclude such individuals from our study. All participants had serum CRP und serum hemoglobin levels within physiological range, and their levels did not differ significantly between groups. Of all the factors examined, only one correlation with ferritin was found in our data set, showing approximately statistically significant power. However, this correlation is roughly 0.33 and it is unlikely that higher numbers of cases would yield higher correlation coefficients. At best, the significance and statistical power would increase. It is striking that other factors that are typically associated with the AHI do not show any further significant correlation with the AHI if one adjusts for the ferritin measurements and their influence. For sleep medicine, the following novelty emerges, which should nevertheless be confirmed in its significance in larger collectives: Compared to CRP, ferritin is a more important laboratory marker for screening populations for the possible presence of a sleep disorder. Especially in healthy populations, sleep history questionnaires should be used to identify risk groups for sleep disorders, especially in the case of selective elevations of ferritin. Therefore, it should be discussed to include serum ferritin not only in the list of surrogate markers for chronic inflammatory diseases but also for OSA. These preliminary findings are quite relevant from a clinical viewpoint, because blood samples could be processed for ferritin even in an outpatient setting and therefore provide general medical practitioners, internal medicine, pulmonary medicine or other (e.g., ear, nose and throat) specialists with a handy surrogate biomarker to assist them in classification of OSA disease severity. Therefore, this could have further potential implications for the necessity and priority of OSA patient management on a personalized way. Additionally, these findings provide the background for further detailed study of the biochemical and molecular mechanisms involved in the interplay of nocturnal intermittent hypoxia and ferritin biochemical functional networks in humans. Of course, further investigations in larger OSA patient cohorts are needed to increase the validity of our preliminary data.

Although a clear positive association between serum ferritin levels and OSA severity was demonstrated, a mechanistic explanation for the possible linkage between OSA and an altered iron metabolism remains unclear. A possible mechanistic explanation could be an increased oxidative stress level of the tissue periphery caused by severe OSA, resulting in an increased serum ferritin level. In this case, not only the pure number of apneic or hypopnea events, but rather the oxygen deficiency could be the causative factor. Previous studies suggest that PLMs are a matter of concern in patients with OSA [28], raising the question of whether elevated PLMs might contribute to elevated serum ferritin levels. However, the presence of PLMs has been associated with low serum ferritin levels [29]. Another explanation for the increased serum ferritin in severe OSA could be due to decreased N3 sleep. N3 sleep is considered the deepest stage of sleep, during which the body repairs and regrows tissues, strengthens the immune system, and builds bone and muscle [30]. The imbalance of sleep stages in severe OSA might contribute to the observed disturbance in iron metabolism. This consideration also applies to the increased ARI in severe OSA, as autonomic activation and sleep fragmentation are relevant consequences of arousals [31]. In contrast, a recent study described lower serum ferritin levels in children with autism spectrum disorders who suffered more frequently from sleep fragmentation [32]. Further studies should be designed to test the value of serum ferritin levels as a biochemical surrogate marker of OSA severity. In due course, prospective replication is needed to test the broader validity of the results of this exploratory analysis.

Our study has certain limitations. First, our study was an observational study, not a randomized controlled trial, which may be considered a limitation. Second, no information was available on lifestyle habits of the participants such as alcohol consumption or cigarette smoking. These stimulants may have altered serum ferritin levels independently. Third, despite careful review of all records, we cannot guarantee that all chronic diseases, current infections, malignancies, or daily or regular use of medications of any kind were accurately reported by the patient at the time the medical record was obtained. Fourth, males constituted the majority of study subjects, especially when OSA severity increased. This sex bias may limit the population to which our results could be applied. Additionally, this sex bias may have altered serum ferritin levels independently. A similar trend has been previously found for CRP levels [33]. In addition, because of the retrospective design, it wasn’t possible to determine which of the included female study participants were premenopausal or postmenopausal. Finally, a remaining potential confounding factor is that serum ferritin levels in our cohort may have been influenced by BMI instead of OSA severity [34]. Future prospective studies with larger samples should focus on the molecular or cellular mechanisms involved.

## 5. Conclusions

We provide evidence that severe OSA may be associated with elevated serum ferritin levels in otherwise healthy OSA subjects. Serum ferritin levels may be a valuable tool for clinicians not only to investigate anemia but also as a surrogate marker of OSA severity, especially in obese individuals.

## Figures and Tables

**Table 1:** Baseline characteristics of the study group.

	None/Mild	Moderate	Severe	Between Group Comparison (*p*-Value)
Number of patients	30	31	29	not significant
Number of female patients (%)	8 (26.7)	7 (22.6)	2 (6.9)	not significant
Age in years (±SD)	42.11 ± 12.99	44.21 ± 11.48	48.06 ± 6.92	not significant
BMI in kg/m^2^ (IQR)	25 (23–27.3)	28 (26–30)	30 (26.5–32.5)	*p* < 0.05 for none/mild vs. moderate, *p* < 0.001 for none/mild vs. severe

“none/mild”—all male and female patients with no or mild form of obstructive sleep apnea (OSA); “moderate”—all male and female patients with moderate form of OSA; “severe”—all male and female patients with severe form of OSA. Abbreviations (in alphabetical order): BMI—body–mass index; IQR—interquartile range. Categorical variables were described as number and percentage (%), and continuous variables were described as mean ± SD for normal distributed or median and IQR for non-normal distributed values.

**Table 2:** Comparison of sleep parameters among the three different patient groups.

	None/Mild	Moderate	Severe	Between Group Comparison (*p*-Value)
AHI in n/h (IQR)	9.3 (6.2–12.7)	23.5 (18.4–26.9)	42.5 (33.5–62.3)	*p* < 0.001 for all groups
AI in n/h (IQR)	1.8 (0.6–3.7)	4.8 (1.9–10.1)	19.3 (11.8–42.3)	*p* < 0.01 for none/mild vs. moderate, *p* < 0.001 for all other groups
HI in n/h (±SD)	6.7 ± 3.3	15.9 ± 5.3	21.6 ± 12.8	*p* < 0.05 for moderate vs. severe, *p* < 0.001 for all other groups
SI in n/h (IQR)	42 (5.2–125.5)	112.8 (34.2–296.2)	264.4 (133.1–416.2)	*p* < 0.001 for none/mild vs. severe
ODI in n/h (IQR)	7 (4.2–11.6)	19.5 (14.3–23.8)	37.6 (28.7–61.7)	*p* < 0.001 for all groups
t90 in % (IQR)	0 (0–0.2)	0.5 (0.1–1)	3.1 (0.8–10.9)	*p* < 0.05 for none/mild vs. moderate, *p* < 0.01 for moderate vs. severe, *p* < 0.001 for none/mild vs. severe
PLM in total (IQR)	15 (5–49)	25 (8.4–129)	78 (14.5–191.5)	*p* < 0.05 for none/mild vs. severe
TST in min (IQR)	374.6 (322.5–399.5)	370.9 (333.6–420.4)	368.2 (326.6–396.1)	not significant
N3 sleep in % (±SD)	18.9 ± 8.9	18.5 ± 9.6	12 ± 6	*p* < 0.01 for none/mild vs. severe, *p* < 0.01 for moderate vs. severe
REM sleep in % (±SD)	17.3 ± 5.5	18.9 ± 5.1	16.1 ± 6.4	not significant
ARI in n/h (IQR)	10.8 (7.5–15.8)	13.1 (10.2–18.2)	21.6 (16.1–29.5)	*p* < 0.001 for none/mild vs. severe, *p* < 0.01 for moderate vs. severe

“none/mild”—all male and female patients with no or mild form of obstructive sleep apnea (OSA); “moderate”—all male and female patients with moderate form of OSA; “severe”—all male and female patients with severe form of OSA. AHI—apnea–hypopnea index; AI—apnea index; HI—hypopnea index; SI—snoring index; ODI—oxygen desaturation index; t90—percentage of oxygen saturation lower than 90%; PLM—periodic limb movements; TST—total sleep time; ARI—arousal index. Further abbreviations (in alphabetical order): IQR—interquartile range; REM—rapid eye movement; SD—standard deviation. Categorical variables were described as number and percentage (%), and continuous variables were described as mean ± SD for normal distributed or median and IQR for non-normal distributed values.

**Table 3:** Comparison of ferritin, C-reactive protein, and hemoglobin serum levels among the three different patient groups.

	None/Mild	Moderate	Severe	Between Group Comparison (*p*-Value)
Serum ferritin level in ng/mL (IQR)	111 (41.8–167)	121 (93–184)	154 (81.5–244)	not significant
Serum CRP level in mg/L (IQR)	1 (0.7–2)	1.5 (0.6–2.8)	1.4 (0.9–2.3)	not significant
Serum hemoglobin level in g/dL (±SD)	14.8 ± 1.2	15 ± 1.2	15.1 ± 1.2	not significant

“none/mild”—all male and female patients with no or mild form of obstructive sleep apnea (OSA); “moderate”—all male and female patients with moderate form of OSA; “severe”—all male and female patients with severe form of OSA. Abbreviations (in alphabetical order): CRP—C-reactive protein; IQR—interquartile range; SD—standard deviation. Continuous variables were described as mean ± SD for normal distributed or median and IQR for non-normal distributed values.

**Table 4:** Different correlations with the apnea–hypopnea index.

x	y	Estimate	Standard Error	t-Value	r	*p*-Value
Age	Log_10_ AHI	0.0090	0.0033	2.6856	0.2767	0.0087
Log_10_ BMI	Log_10_ AHI	1.2309	0.4758	2.5869	0.2673	0.0113
Log_10_ Ferritin	Log_10_ AHI	0.3507	0.1098	3.1943	0.3240	0.0020
Log_10_ CRP	Log_10_ AHI	0.2152	0.1008	2.1345	0.2231	0.0356
Log_10_ Ferritin (adjusted for age)	Log_10_ AHI	0.2987	0.0987	3.0274	0.3639	0.0032

Abbreviations (in alphabetical order): AHI—apnea–hypopnea index; BMI—body–mass index; CRP—C-reactive protein.

## Data Availability

The data presented and analyzed in this study are available on reasonable request from the corresponding author.
